# Exploring the Relationship Between Hearing Loss and Cognitive Dysfunction from the Perspective of Molecular Mechanisms

**DOI:** 10.3390/biom16070986

**Published:** 2026-07-04

**Authors:** Lu Wang, Ziqi Zhou, Xingqian Shen, Guolei Wu, Xiaoye Chen, Bo Liu, Hongjun Xiao

**Affiliations:** 1 Department of Otorhinolaryngology-Head and Neck Surgery, ENT Institute, Union Hospital, Tongji Medical College, Huazhong University of Science and Technology, Wuhan 430022, China; 2 Clinical Medical Research Center of Deafness and Vertigo in Hubei Province, Wuhan 430022, China

**Keywords:** hearing loss, cognitive dysfunction, molecular mechanism, tau protein

## Abstract

Dementia is primarily characterized by cognitive dysfunction, which is one of the major causes of disability. As hearing loss has been identified as the largest modifiable risk factor for dementia, understanding the intrinsic relationship between the two is crucial for preventing and mitigating the progression of dementia. A body of research has demonstrated a close association between hearing loss and morphological changes in the brain; however, the underlying molecular mechanisms require further elucidation. This review synthesizes current evidence on tau protein, amyloid beta protein, glutamate, gamma-aminobutyric acid, reactive oxygen species, and brain-derived neurotrophic factor to clarify potential molecular pathways linking hearing loss with cognitive dysfunction and to identify candidate targets for future mechanistic and translational studies.

## 1. Introduction

Hearing loss (HL) is defined as a sensory impairment characterized by reduced hearing capacity, which manifests as the inability to hear as well as someone with normal hearing. Normal hearing typically refers to hearing thresholds of 20 dB or better in both ears [1]. An estimated 1.57 billion people worldwide were affected by HL in 2019, representing one-fifth of the global population. Among them, 62.1% were over the age of 50. By 2050, it is projected that 2.45 billion individuals will suffer from HL, reflecting a 56.1% increase compared to 2019 [2]. Age-related sensorineural hearing loss (ARHL) is the most prevalent form among adults and is defined as a progressive, bilateral, and symmetrical sensorineural hearing loss associated with aging, which is most pronounced at higher frequencies [3,4,5].

The primary consequence of HL is impaired communication ability, which can adversely affect interpersonal relationships and lead to occupational difficulties. It also has indirect consequences on health, psychosocial well-being, and economic status [6]. The Lancet Commission has identified HL as one of the potentially modifiable risk factors for dementia [7]. Dementia is generally defined as a progressive cognitive dysfunction that results in loss of independent functioning [8]. Its burden is significant at individual, community, national, and global levels, with Alzheimer’s disease (AD) being its most common cause [9,10]. However, dementia-related cerebral changes can cause central auditory dysfunction, impairing processes such as the segregation of target signals from background noise—a critical function for daily hearing [11,12]. Thus, although HL is a risk factor for dementia, dementia-related neuropathological alterations may exacerbate auditory impairment. Therefore, identifying appropriate targets and implementing timely interventions are essential to mitigate the progressive deterioration of both hearing and cognitive function.

Numerous studies have investigated the association between HL and brain structural changes. Koops et al. observed that the HL group without tinnitus exhibited reduced gray matter volume and cortical thickness in several brain regions both within and outside the auditory pathway [13]. In the study by Ren et al., individuals with ARHL showed cortical atrophy in multiple auditory cortical areas and default mode network (DMN) nodes compared to age-matched normal-hearing controls. These structural alterations in DMN nodes were correlated with cognitive impairment in ARHL patients [14]. Armstrong et al. found that poorer hearing in midlife was associated with steeper volume decline in later life in the right temporal lobe gray matter, right hippocampus, and left entorhinal cortex—regions affected in the early stages of AD [15]. Parker et al. reported that peripheral HL could predict a faster rate of global brain atrophy, which in turn was associated with greater cognitive decline [16]. These findings highlight the potential impact of HL on brain morphology and cognitive function.

Furthermore, several studies have investigated the potential molecular mechanisms linking HL and cognitive function; however, their findings are inconsistent and subject to ongoing debate. Moreover, there is a scarcity of comprehensive reviews that systematically summarize and elucidate the current understanding in this field, which poses certain challenges for the exploration of relevant molecular targets.

Although HL is widely recognized as a risk factor for cognitive impairment, the current evidence regarding the use of hearing aids (HAs) to mitigate cognitive impairment remains inconsistent. In a study by Grenier et al., the researchers found that the severity of HL was associated with the risk of cognitive impairment, but they did not observe any significant benefit from the use of HAs in the general population [17]. In contrast, a meta-analysis by Yeo et al. found that the use of HAs significantly reduced the risk of cognitive decline [18]. The ACHIEVE randomized controlled trial found that the effectiveness of hearing intervention was associated with the participants’ risk of cognitive decline; whilst a significant effect of hearing intervention in slowing the rate of cognitive decline was observed in high-risk individuals, no such effect was observed in low-risk individuals [19]. Recently, Jiang et al. conducted a meta-analysis of seven longitudinal aging cohorts and found that the use of HAs was associated with a reduced risk of dementia; however, this association was limited to participants who reported that their hearing had improved as a result of the intervention [20].

These discrepancies may stem from differences in participants’ health status, the effectiveness of HAs, socioeconomic status, and other characteristics, or from a possible non-linear relationship between HL and cognitive impairment. Therefore, further investigation into the biological links between HL and cognitive impairment, and the underlying mechanisms, is of significant clinical importance. This provides important insights into the development of new diagnostic and therapeutic approaches. Elucidation of the molecular mechanisms linking HL and cognitive dysfunction could enable the use of relevant molecules as early biomarkers. These biomarkers would help identify individuals at high risk for cognitive decline and facilitate early diagnosis. Moreover, developing targeted drugs based on these mechanisms could potentially decelerate the progression of cognitive impairment. If a third factor simultaneously influences both hearing and cognitive function, then therapeutic strategies targeting these common pathways hold significant clinical potential for simultaneously mitigating HL and cognitive decline. This review aims to provide theoretical directions for future in-depth investigations into the molecular mechanisms connecting HL and cognitive function, as well as provide potential targets and theoretical foundations for molecular-targeted therapies.

**Figure 1:**
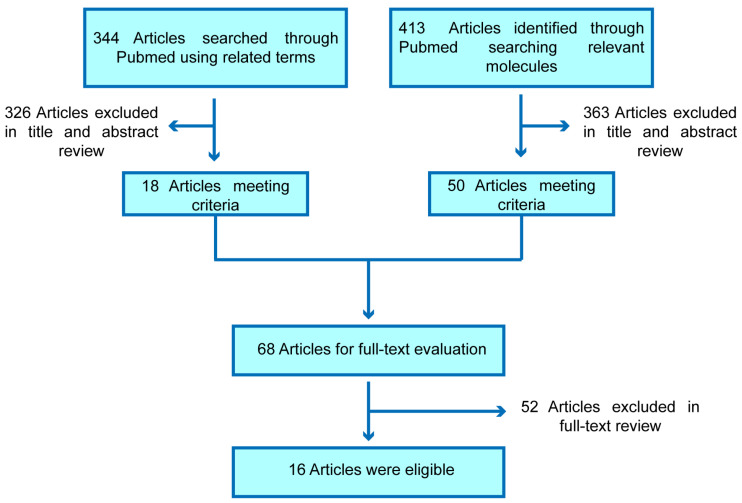
Flow diagram of the literature search and study selection process.

As shown in Figure 1, we conducted a literature search on PubMed using combinations of the following terms: “hearing loss,” “hearing impairment,” “deafness,” “hypoacusis,” “presbycusis,” “cognitive dysfunction,” “cognitive decline,” “cognitive disorder,” “cognitive impairment,” “molecular mechanism,” “pathogenesis,” “pathway,” “molecular pathway,” and “biomarker,” to identify articles published between January 2015 and October 2025. A total of 344 articles were retrieved. The primary literature screening was performed by two members of the research team (L.W. and X.S.). By reviewing the titles and abstracts, we included only those articles focusing on molecules involved in the relationship between HL and cognitive dysfunction. A total of 18 articles met the criteria. Additionally, we performed supplementary searches by combining HL with molecules closely associated with cognitive dysfunction, including tau protein, amyloid beta-protein (Aβ), glutamate (Glu), gamma-aminobutyric acid (GABA), reactive oxygen species (ROS), neurotrophic factors, dopamine, vasopressin, and somatostatin. These searches identified 413 articles. After initial screening of titles and abstracts and excluding articles already retrieved, 50 articles proceeded to full-text review. Following independent reviews conducted by the two members, 16 articles were ultimately selected: one on Aβ and tau, five on tau, four on Aβ, 3 on Glu, one on Brain-derived neurotrophic factor (BDNF), and two on ROS. We summarized and synthesized the findings from these articles to elucidate the molecules potentially involved in the relationship between HL and cognitive dysfunction, as well as the underlying mechanisms.

## 2. Tau Protein May Play a Mediating Role

Tau protein, discovered in 1975, was one of the first characterized microtubule-associated proteins. It is primarily expressed in neurons, facilitating the assembly of microtubules and stabilizing the microtubule network. Tau protein also plays a role in regulating the integrity of the axon initial segment, maintaining neuronal excitability homeostasis, and supporting nuclear maintenance [21]. Abnormal phosphorylation of tau protein reduces its affinity for microtubules and facilitates its aggregation into neurofibrillary tangles (NFTs), leading to a loss of normal function and tau-mediated neurotoxicity [22]. This process is one of the hallmark pathological features of AD [23].

Recent studies have increasingly examined tau pathology as a potential mediator of HL-associated cognitive dysfunction. We summarize the key findings of these studies in Table 1. Xu et al. reported higher baseline tau and phosphorylated tau 181 (p-tau181) levels in cerebrospinal fluid (CSF), as well as faster increases in these biomarkers, among patients with ARHL [24]. This finding suggests that the abnormal expression of tau protein may not be a mere concomitant phenomenon; its dynamic changes might have a closer temporal correlation with the progression of ARHL. Shen et al. observed that the level of p-tau increased over time, but total tau (t-tau) expression was unchanged in a mouse model of severe bilateral HL induced by kanamycin and furosemide [25]. This may be attributed to the earlier elevation of p-tau protein compared to t-tau [26]. Furthermore, given that p-tau exhibits a close correlation with tau pathology in the brain, p-tau could potentially serve as a more sensitive biomarker for HL-induced cognitive dysfunction [27]. Wang et al. reported that hearing impairment was linked to a higher level of CSF tau protein, with mediation analyses suggesting that tau pathology partly mediated the association between hearing impairment and cognitive decline [28]. This supports that tau pathology may be one of the factors mediating HL-induced cognitive dysfunction, although other mechanisms may be involved in this process. Zheng et al. demonstrated that worsening hearing was correlated with increased burden of tau protein detected by positron emission tomography (PET) [29]. The imaging evidence provides a valuable complement to molecular biological findings.

However, some studies have found no association between tau protein and HL. Martínez-Dubarbie et al. reported that no statistically significant correlations were found between p-tau181 levels and any of the objective auditory measures. The participants’ inclusion criteria for this study were more stringent, enrolling only cognitively unimpaired older adults [30]. Specifically, we aimed to identify molecules associated with both HL and cognitive dysfunction; therefore, our targeted population may represent a later stage. Katanga et al. found that neurofibrillary tangle staging (based on the distribution and sequential progression of abnormally p-tau-induced NFTs) within the brain and overall AD neuropathology level were not significantly associated with an increased risk of HL. Because HL was assessed using self-reported outcomes rather than objective audiometric measures, some participants may not have been aware of their hearing difficulties, potentially leading to misclassification bias [31].

Most available studies support an association between tau pathology and HL. Thus, tau protein is likely involved in the relationship between HL and cognitive dysfunction, although the underlying mechanisms require further investigation.

Griffiths et al. proposed that increased auditory cognitive processing in response to degraded input elevates neuronal activity in the medial temporal lobe (MTL), thereby potentially promoting AD-related pathology and accelerating cognitive decline. MTL, which comprises structures such as the hippocampus, entorhinal cortex, and amygdala, serves as a central hub for memory encoding, spatial cognition, and sensory information integration [32]. As noted above, enhanced neuronal activity in the MTL may mediate the relationship between HL and tau protein. Shen et al. found in a mouse model that HL increased p-tau levels, which were associated with hippocampal neuroinflammation [25]. This may suggest that neuroinflammation is associated with both HL and elevated p-tau and may play a mediating role in their relationship; however, further research is needed to validate these findings. Chen et al. also showed that the pathological activation of glial cells and the release of inflammatory factors during the process of neuroinflammation can aggravate tau pathology through direct or indirect pathways [33]. Furthermore, tau pathology has been shown to directly activate microglia and disrupt their homeostasis, potentially aggravating neuroinflammation [34]. Based on these findings, we hypothesize that neuroinflammation participates in mediating HL-induced tau pathology, while tau pathology further exacerbates neuroinflammation, thereby aggravating cognitive dysfunction. However, the translation of these findings from animal models to human mechanisms warrants further investigation.

Brain regions involved in both auditory processing and memory, such as MTL, may be particularly relevant to this mechanism. Tau deposition in these regions could contribute to the progressive deterioration of both cognitive and auditory functions during disease progression [35]. Synthesizing the aforementioned perspective of Griffiths et al., the accumulation of tau protein in the MTL could further impair cognitive and auditory functions, which may present one of the mechanisms underlying the progressive deterioration of both cognitive and hearing during the disease course. Based on the discovery that tau protein can be transported between cells, Zhang et al. proposed that HL may promote p-tau deposition, which subsequently spreads from cognitive regions to the auditory region, thereby further exacerbating auditory impairment [36]. Therefore, we hypothesize that the deposition of tau protein in auditory-related brain regions may be involved in the intrinsic mechanism underlying the progressive deterioration of ARHL.

Alberti et al. found that participants with HL exhibited significantly higher p-tau181 levels compared to matched control groups. After the fitting of HAs, auditory, cognitive, and serum biomarkers improved [37]. This study suggests that improving hearing through the use of HAs may mitigate tau pathology and cognitive decline, which may provide further support for the association between HL and tau protein. However, the verbal components of cognitive tests in this study may be associated with an underestimation of the cognitive level. Given the limited number of similar studies, the effect of HAs on tau pathology requires further validation.

**Figure 2:**
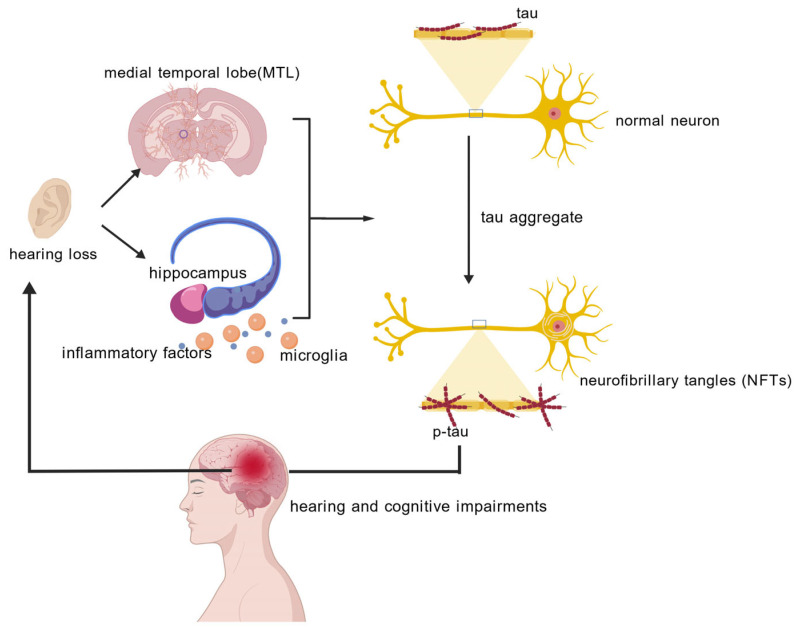
Hypothetical diagram of the mechanism underlying tau protein-mediated association between hearing loss and cognitive dysfunction (Created with BioGDP.com; Created in BioRender. Zhou, Z. (2026) https://BioRender.com/viu1u7z, accessed on 6 May 2026).

In summary, available evidence supports an association between HL and tau pathology, although the causal mechanisms remain unresolved. We hypothesize that HL may be linked to cerebral tau deposition through mechanisms such as increased neuronal activity in MTL and neuroinflammation in the hippocampus. Conversely, tau accumulation in auditory-related brain regions could potentially exacerbate ARHL (Figure 2). The application of HAs may improve auditory function, which might consequently reduce tau protein deposition and enhance cognitive performance. However, while several studies reported positive associations, the strength of evidence in these studies—whether their findings are positive or negative—is limited by several factors: small sample sizes, the current lack of standardized measurements for auditory function and tau protein levels, the inherent variability in tau protein levels, and the potential influence of comorbidities [30,38]. Therefore, the possibility of a null association cannot currently be excluded.

## 3. The Effect of Amyloid Beta-Protein Is Influenced by Other Factors

The amyloid cascade hypothesis has become the primary model for the pathogenesis of AD. According to the hypothesis, the accumulation of toxic Aβ in the central nervous system (CNS) is considered the main cause of AD. The aggregation of Aβ into plaques exerts neurotoxicity and contributes to dementia through cytopathic effects. Aβ peptides are generated via proteolytic cleavage of the amyloid precursor protein (APP), a type I transmembrane glycoprotein widely expressed in tissues and particularly enriched at neuronal synapses. APP processing occurs through two principal pathways: the non-amyloidogenic pathway and the amyloidogenic pathway. In the amyloidogenic pathway, APP is sequentially cleaved by β- and γ-secretases, resulting in the production of Aβ peptides of varying lengths [39,40].

Given the pivotal role of Aβ in AD pathogenesis, several studies have examined its association with HL, with the relevant studies summarized in Table 2. In a longitudinal cohort study utilizing the ADNI database, Xu et al. found no correlation between the ARHL and Aβ levels measured via CSF or PET scans. Furthermore, they observed that ARHL did not affect the CSF Aβ1-42/Aβ1-40 ratio and concluded that this lack of association could not be attributed to inter-individual variations in total Aβ production [24]. Deal et al. conducted a cross-sectional study that enrolled elderly individuals with a mean age of 81 years, and they found no association between hearing thresholds and cortical or temporal Aβ deposition, as measured by standardized uptake value ratios (SUVRs) from PET scans. Their findings indicated no link between HL and Aβ burden in the cortex, including the temporal lobe—a critical region involved in both auditory and cognitive functions—providing further evidence against a significant association [41]. Similarly, Sarant et al. reported that while both HL and Aβ levels increased with age, no correlation remained after controlling for age, suggesting that age may be a confounding factor in the relationship between HL and Aβ [42].

Notably, discrepancies in findings have emerged across studies involving different participant populations, which may point to meaningful underlying factors. In a cross-sectional study by Golub et al., a significant relationship was observed between pure-tone average (PTA) and whole-brain Aβ SUVRs, with a 10 dB increase in PTA corresponding to a 0.029 increase in SUVRs [43]. They included younger participants in this study, which may partially account for the discrepancy with the aforementioned research findings. Considering that the age groups of the studies yielding negative associations were generally higher than that of the current study, we aimed to further explore whether Aβ exerts differential effects depending on age group.

Different from the findings of Sarant et al., who reported that both the degree of HL and Aβ deposition increase with age, Van’t Hooft et al. reported that HL was associated with higher amyloid burden in younger-old groups but not in oldest-old individuals. They proposed that the mechanisms linking HL to dementia risk may vary with age [44]. In younger-old individuals, hearing impairment was associated with AD pathology in brain structures involved in both auditory processing and cognition, supporting the common cause hypothesis. In contrast, among the oldest-old adults, the observed association between auditory function and cortical atrophy aligned with the sensory deprivation hypothesis, which posits that long-term HL induces neuroplastic changes, although reduced sensory input alone was insufficient to explain structural brain changes [44]. This may suggest that the association between HL and cognitive impairment across different age groups involves distinct neuropathological processes rather than a single causal mechanism. Based on this perspective, HL may be more closely associated with Aβ deposition in younger-old adults than in the oldest-old population. In addition to the common factors discussed by Van’t Hooft, several animal studies have also demonstrated an association between HL and Aβ deposition. Although direct extrapolation of these pathways is limited by differences between animal models and human disease, these mechanisms may still provide valuable clues for exploring the corresponding processes in humans. In an animal model, Ko et al. suggested that HL may initiate neuroinflammatory processes that subsequently promote Aβ deposition [45]. Pan et al. showed that HL led to the downregulation of embryonic growth/differentiation factor 1 (GDF1) in the hippocampus. This subsequently activated asparagine endopeptidase (AEP), which functions as a δ-secretase and promotes Aβ production [46]. In the oldest-old adults, the mediating role of Aβ may become less predominant, while other mechanisms could play a relatively greater role.

Therefore, we hypothesize that the role of Aβ in the relationship between HL and cognitive impairment may be influenced by factors such as age. This could partly explain the substantial heterogeneity observed in studies investigating the association between HL and Aβ. Based on the currently available evidence, Aβ does not appear to be a consistently robust mediator linking HL to cognitive decline. Although the perspective that age may modulate the mechanisms underlying Aβ’s effects offers novel insights, relevant studies are still scarce and insufficient to exclude the presence of alternative explanations.

## 4. Imbalance Between Glutamate and Gamma-Aminobutyric Acid as a Potential Mediator

Glu is the primary excitatory neurotransmitter in the CNS, while GABA serves as the major inhibitory neurotransmitter. Both play crucial roles as the principal neurotransmitters in the auditory system. Inhibitory GABA is synthesized from Glu via glutamate decarboxylase and modulates glutamatergic excitation [47]. The balance between excitation and inhibition is essential for maintaining homeostasis in the CNS, and disruptions in this equilibrium are associated with various neurological disorders [48]. Some studies have suggested a link between HL and altered levels of GABA and Glu, prompting further investigation into whether these neurotransmitters mediate the association between HL and cognitive dysfunction [49,50].

Qiu et al. proposed that a reduction in the degree of cortical folding in the right Brodmann area 52, a region within the auditory network, plays a significant mediating role between hearing ability and executive function, and that this mediating effect is modulated by altered levels of Glu in the right auditory region [51]. Given the crucial role of Glu in regulating neurogenesis, synaptogenesis, neurite outgrowth, and neuronal survival in the brain, it is plausible that reduced Glu levels associated with HL may disrupt normal neurophysiological processes, potentially contributing to decreased cortical folding [52]. Correspondingly, in a mouse model of noise-induced hearing loss, Hayes et al. found that the expression of the NR2B subunit of N-methyl-D-aspartate (NMDA) was up-regulated in the auditory cortex [53]. NMDA is one of the Glu receptors and plays a significant role in long-term potentiation and long-term depression, which are closely associated with memory and learning [52]. This upregulation may represent a compensatory response to altered glutamatergic signaling following HL. Beyond Glu, Li et al. found that ARHL leads to decreased GABA levels in the right auditory cortex. Local GABAergic interneurons are known to play an important role in modulating synchronous activity between distant brain regions. The decline in GABA levels reduces functional connectivity between the auditory network and the DMN, potentially indicating a disruption in the link between auditory and cognitive processing [54]. Su et al. proposed that HL might disrupt excitation-inhibition (E/I) balance (measured by the Glu/GABA ratio) in the right auditory cortex. The E/I balance is a critical determinant of network stability, and its disruption may mediate the compensatory mechanisms of whole-brain dynamic functional networks. Maladaptive exhaustion of inter-network compensatory mechanisms could ultimately lead to cognitive dysfunction [55].

We hypothesize that ARHL may alter GABA and Glu levels in the auditory cortex, resulting in an imbalance between excitation and inhibition. Reduced meaningful stimulation might shift the E/I balance toward excitation due to compensatory mechanisms [55]. In the auditory cortex, disruptions in Glu and GABA levels, along with the resulting E/I imbalance, can have corresponding detrimental effects on normal neurophysiological processes, leading to cognitive dysfunction.

We hypothesize that such changes may be associated with HL-induced alterations in the canonical ascending auditory pathway. In addition to this pathway, the reticular-limbic pathway is also involved in auditory signal transmission and is considered to be related to emotions, arousal, attention, and memory. Liu et al. identified a mechanism involving the reticular-limbic pathway, showing that HL affects the transmission from glutamatergic neurons of the caudal pontine reticular nucleus to norepinephrinergic neurons of the locus coeruleus, thereby influencing norepinephrine levels, which are associated with neurogenesis in the hippocampus [56]. Therefore, we propose that HL may play a role in neurotransmitter changes in both the canonical ascending auditory pathway and the reticular-limbic pathway, thereby leading to structural or functional alterations associated with cognitive dysfunction. However, whether HL induces changes in Glu and GABA levels in the auditory cortex through alterations in the canonical ascending auditory pathway remains unclear. Moreover, because most available studies are cross-sectional, the specific contributions of Glu and GABA to both HL and cognitive dysfunction remain difficult to determine.

## 5. Oxidative Stress Induced by Reactive Oxygen Species as a Common Factor

ROS are byproducts of cellular aerobic metabolism, including superoxide anion, hydrogen peroxide, hydroxyl radical, and nitric oxide. Mitochondrial respiration is a major source of ROS [57,58]. Antioxidant enzymes scavenge free radicals to regulate ROS levels [59]. While a lower concentration of ROS is essential for normal cellular signaling, excessive ROS disrupts the balance between oxidants and antioxidants, leading to oxidative stress. This can damage macromolecules such as DNA, lipids, and proteins, ultimately resulting in cell necrosis and apoptosis [60,61]. Due to their high oxygen consumption, high lipid content, abundant mitochondria, and substantial energy demands, neurons and hair cells are particularly vulnerable to ROS-induced damage [62].

In the cochlea, ROS can damage DNA, break down lipids and proteins, and trigger cochlear cell apoptosis [58]. Mitochondria-derived ROS may also cause oxidative damage to mitochondrial DNA (mtDNA), inducing mitochondrial dysfunction and further accelerating ROS production. This exacerbates cochlear cell apoptosis and the progression of HL [63].

Pisani et al. provided support for the role of oxidative stress in HL. They used the C57BL/6 mouse model, a widely used model of ARHL, and found that elevated levels of 4-hydroxynonenal (4-HNE), 3-nitrotyrosine (3-NT), and tumor necrosis factor-alpha (TNF-α) in cochlear tissues were closely associated with the progression of HL. Increased levels of 4-HNE and 3-NT indicate the occurrence of nitrosative stress and lipid peroxidation, while elevated TNF-α highlights the contribution of inflammation. These findings support that oxidative stress and inflammation synergistically promote cochlear degeneration [64]. Du et al. conducted a study using D-galactose-induced aged mice, a common model in aging research. In the cochlear mitochondria of these mice, they observed a marked increase in 8-hydroxy-2-deoxyguanosine (8-OHdG), a marker of DNA oxidative damage, along with decreases in superoxide dismutase activity and ATP production. They proposed that oxidative damage to mtDNA, followed by dysfunction of inner hair cells and spiral ganglion cells, may cause damage to the presynaptic and postsynaptic structures of the cochlear ribbon synapse. They supported that the cochlear ribbon synapse is the primary site of early damage in ARHL [65].

Oxidative stress is closely linked to neurodegenerative diseases and aging. There exists a complex interplay between oxidative stress and the deposition of Aβ and NFTs. Aβ interacts with redox-active metals (e.g., copper, zinc, and iron) to form complexes. This interaction promotes both Aβ aggregation and ROS generation [60]. Additionally, Aβ can bind to the mitochondrial membrane, leading to energy metabolism disorders and subsequently promoting ROS production. Excessive ROS, in turn, is associated with enhanced abnormal processing of APP into Aβ [59]. ROS can induce lipid peroxidation in neuronal membranes, producing byproducts such as 4-HNE. 4-HNE then induces conformational changes in the tau protein, ultimately promoting its aggregation into NFTs [66]. P-tau affects Complex I activity, thereby influencing mitochondrial function and ROS generation [59]. Thus, ROS, Aβ, and tau pathology may interact reciprocally, forming a self-reinforcing pathological cycle.

In the study by Shin et al., it was found that 5XFAD transgenic mice, one of the most widely used animal models of AD characterized by Aβ plaques and cognitive impairment, exhibited poorer performance in a prefrontal cortex-dependent learning set-shifting task compared with control mice. In addition, these mice showed higher levels of 4-HNE in the prefrontal cortex [67]. Mecocci and colleagues observed that levels of 8-OHdG in both nuclear DNA and mtDNA increased with age, with a faster rate of accumulation in mtDNA. They also measured 8-OHdG levels in the brains of AD patients and found a significant increase in mtDNA of the temporal cortex compared with controls [68]. Their findings provide support for the occurrence of oxidative damage during AD pathogenesis, with mtDNA being particularly susceptible, and suggest a possible association between such damage and cognitive performance.

We propose that ROS may play a critical role in the pathogenesis of HL and various neurodegenerative disorders. Shen et al. also suggest that aging typically leads to reduced ROS clearance and enhanced ROS activity, resulting in intracellular ROS accumulation. This accumulation causes mtDNA mutations and mitochondrial dysfunction, which in turn reduce vascular endothelial growth factor (VEGF) levels. VEGF plays a vital role in angiogenesis and neuronal protection, and its decline may contribute to Aβ deposition, impaired repair of hair cells and spiral ganglia, and compromised cerebrovascular remodeling [69].

We posit that age-related ROS accumulation may significantly contribute to the pathogenesis of ARHL and neurodegenerative diseases. Oxidative stress damages cellular molecules, leading to cochlear and neuronal cell injury. Furthermore, it mutually exacerbates the deposition of Aβ and NFTs. This mechanism may represent a shared etiological factor in both HL and cognitive dysfunction.

Nevertheless, it should be noted that current studies simultaneously addressing oxidative stress, HL, and cognitive impairment are very limited, making it difficult to clearly understand the complex relationships among the three. Although oxidative stress is a common feature of aging, its precise role in the association between HL and cognitive impairment remains unclear. Future studies should determine whether oxidative stress acts as a shared etiological factor, mediator, or modifier in this relationship.

## 6. Reduced Levels of Brain-Derived Neurotrophic Factor as a Shared Mechanism

BDNF is a key regulator of neural circuit development and function [70]. Initially synthesized as a precursor protein (pro-BDNF), BDNF can be cleaved by various proteases to yield mature BDNF (m-BDNF). Pro-BDNF and m-BDNF interact with distinct receptors and exert opposing effects on cellular functions: pro-BDNF induces long-term depression and apoptosis, whereas m-BDNF activates signaling pathways that promote cell survival and proliferation. Under physiological conditions, BDNF exerts protective effects in the hippocampus by enhancing long-term potentiation, synaptic plasticity, and neuronal survival [71]. It also plays a crucial role in the development of the inner ear, including the development and maintenance of spiral ganglion neurons [72].

Altay et al. found that plasma BDNF levels were significantly decreased in patients with ARHL compared with age-matched controls [73]. Zhang et al. conducted a case–control study and reported that lower plasma BDNF levels were associated with the presence of mild cognitive impairment in patients with AD, and they suggested that plasma BDNF levels may be associated with preserved cognitive function [74]. However, plasma BDNF may originate from multiple sources, and its level changes could be influenced by other factors. De Vries et al. provided supporting evidence at the gene expression level, showing that BDNF expression is altered in patients with HL and correlates with the severity of hearing impairment [75]. Van den Bosch et al. also found that the BDNF Val66Met genotype significantly amplifies the negative impact of Aβ pathology on cognition, and that this polymorphism is associated with reduced BDNF secretion in the hippocampus [76].

These research findings suggest that altered BDNF levels may be involved in the development of HL or cognitive dysfunction. The potential role of BDNF in the pathogenesis of these two conditions is discussed below.

In AD, an altered pro-BDNF/m-BDNF ratio leads to the accumulation of pro-BDNF, which activates pathways involved in apoptosis and long-term depression, thereby promoting neuronal damage [71]. As AD progresses, the levels of BDNF decline, thereby compromising its protective effects under physiological conditions. Moreover, a detrimental feedback loop may exist between reduced BDNF levels and the accumulation of AD pathological markers, such as Aβ aggregates and hyperphosphorylated tau. BDNF can regulate APP processing and the process of tau phosphorylation and distribution. However, Aβ oligomers not only impair BDNF signaling but also significantly suppress BDNF expression. Similarly, tau overexpression or hyperphosphorylation reduces the expression of BDNF [77].

When cochlear damage occurs, pro-BDNF binds to the P75 neurotrophin receptor, activating downstream signaling proteins that ultimately lead to cell death [71]. BDNF is expressed by hair cells and supporting cells of the organ of Corti. Damage to these cells in the auditory epithelium results in decreased BDNF expression, contributing to degenerative changes in spiral ganglion cells [72].

Under physiological conditions, BDNF plays a vital protective role in both the hippocampus and the inner ear. Altered BDNF levels are also implicated in the pathogenesis of HL and cognitive dysfunction.

However, this perspective has been developed largely from studies that examined the association of BDNF with either HL or cognitive impairment separately, while research simultaneously investigating the relationships among all three remains very limited. Therefore, theoretical extrapolation based solely on these individual studies is insufficient. Further research is needed to simultaneously assess BDNF, auditory function, and cognitive outcomes.

## 7. Limitations and Future Perspectives

Based on existing studies, this review summarizes the potential molecular mechanisms linking HL and cognitive impairment. However, given that most current studies are observational and cross-sectional in design, we are unable to establish causal relationships. Moreover, the lack of standardized audiometric and cognitive assessment tools across studies introduces heterogeneity. Confounding factors such as age and education level may also influence the observed effects of the investigated molecules. Additionally, translational limitations exist between animal studies and human clinical research. Furthermore, when discussing the roles of oxidative stress and BDNF, the limited number of studies examining the interrelationships among these molecules, HL, and cognitive dysfunction constrains our ability to determine the underlying mechanisms. We anticipate that future in-depth research in this field will address these issues, and interventional studies are warranted to further elucidate the roles of relevant molecules.

Beyond the molecules that have received considerable attention as discussed earlier, other mechanisms, including neuroinflammatory cytokines, hippocampal m6A RNA methylation, cerebrovascular dysfunction, and genetic overlap between HL and AD, may also contribute to the association between HL and cognitive impairment. Factors such as physical frailty, depression, education level, social isolation, socioeconomic status, and common neurodegenerative pathologies may also influence the relationship. Future in-depth investigations into these mechanisms may yield significant insights. Furthermore, this review focused solely on peripheral HL and did not explore the relationship between central HL and cognitive function. Future investigations into the potential mechanisms underlying central HL and cognitive impairment may provide a more comprehensive perspective for research on the association between hearing and cognition.

By summarizing the potential molecular links between HL and cognitive impairment, this review aims to facilitate the identification of novel therapeutic targets. Some potential agents have already been explored in existing studies.

Marie and colleagues utilized the SAMP8 mouse model, which exhibits progressive age-related brain dysfunction and progressive HL. They found that oral administration of N-acetylcysteine (NAC), a potent antioxidant, alleviated ARHL and memory decline [78]. 7,8-Dihydroxyflavone (DHF), a high-affinity tyrosine kinase receptor B (TrkB) agonist that mimics the effects of BDNF, has been reported to ameliorate AD-related cognitive deficits [79]. Kempfle et al. introduced a bisphosphonate DHF conjugate. In their in vitro experiment, they demonstrated that inner ear delivery of this conjugate preserved the ability of DHF to support the growth of spiral ganglion neurons, suggesting a novel approach for targeted drug delivery in the treatment of sensorineural hearing loss [80].

These findings suggest that NAC and DHF may serve as potential therapeutic options for HL and cognitive impairment. Nevertheless, clinical trial results remain controversial, and their therapeutic benefits warrant further investigation.

In addition to animal studies, the effects demonstrated by certain drugs in clinical trials also provide us with promising leads. Clinical data from Foster et al. indicate that OTO-413, a sustained-release formulation of BDNF intended for intratympanic administration, produces clinically significant improvements in speech comprehension in patients with HL [81]. Polyphenols are a class of plant-derived bioactive compounds with potent antioxidant properties. A meta-analysis based on 13 randomized controlled trials indicates that polyphenol supplementation is associated with significant improvements in overall cognitive function; however, this protective effect is specific to particular polyphenol subclasses, with flavonoids primarily enhancing cognitive performance, whilst stilbenes predominantly support daily functioning [82]. Nevertheless, clinical research on the effects of antioxidants on hearing improvement remains limited and heterogeneous [83].

Regarding other medications used for dementia, such as memantine, studies on their efficacy in preventing and treating HL-related cognitive impairment are still scarce, and future research should explore this area in greater depth.

## 8. Conclusions

As the population ages, ARHL and cognitive dysfunction are likely to become more prevalent. Several hypotheses have been proposed to explain the potential relationship between HL and cognitive dysfunction, including the cognitive load hypothesis, information degradation hypothesis, sensory deprivation hypothesis, and common cause hypothesis. However, no single hypothesis appears sufficient to fully explain the complex association between HL and cognitive impairment. The available evidence supports a multi-pathway association. Age-related accumulation of ROS and reduced levels of BDNF may represent shared pathological processes. Tau pathology may contribute to this association through altered neuronal activity and neuroinflammation, whereas Glu/GABA imbalance may affect neural network stability. Aβ may not be the primary influencing factor, and the related mechanisms may be affected by multiple factors, such as age.

## Figures and Tables

**Table 1:** Summary of studies on the relationship between HL and tau protein.

Author	Research Objects	Sample Size (N)	Indicators of Hearing	Indicators of Tau Protein	Results
Xu et al., 2019 [24]	Participants from ADNI	479	MH and PE datasets	t-tau and p-tau in CSF	ARHL was related to t-tau and p-tau in CSF, along with a faster rate of elevation of these biomarkers
Shen et al., 2021 [25]	C57BL6 mice	84	ABR	t-tau and p-tau in the hippocampus	The level of p-tau increased over time but t-tau expression was unchanged after HL
Wang et al., 2022 [28]	Participants from UK, CABLE, or ADNI	UK: 165,550 CABLE: 863 ADNI: 1770	SiN, questionnaire, MH and PE datasets	t-tau and p-tau in CSF	HL was correlated with a high level of CSF tau protein in CABLE and ADNI
Zheng et al., 2022 [29]	Elderly from China	57	PTA and WRS	tau PET imaging	ARHL has a significant relationship with tau SUVRs
Martínez-Dubarbie et al., 2024 [30]	Cognitively normal elderly from Spain	55	PTA, WRS, SRT, BHL, HHI-E, and S-AIADH	p-tau in CSF	No statistically significant correlations between p-tau levels and any of the objective auditory measures
Katanga et al., 2023 [31]	British autopsy samples	442	observer-rated assessment	Braak NFT stage	NFT staging was not clearly associated with increased risks of HL

Note: HL: hearing loss; ADNI: Alzheimer’s Disease Neuroimaging Initiative database; MH: medical history; PE: physical exam; t-tau: total tau; p-tau: phosphorylated tau; CSF: cerebrospinal fluid; ARHL: age-related hearing loss; ABR: auditory brainstem response; UK: UK biobank; CABLE: Chinese Alzheimer’s Biomarker and Lifestyle study; SiN: speech-in-noise test; PTA: pure-tone average; WRS: word recognition score; PET: positron emission tomography; SUVRs: standardized uptake value ratios; SRT: speech recognition threshold; BHL: Bilateral Hearing Loss; HHI-E: hearing handicap inventory for the elderly; S-AIADH: Spanish version of the Amsterdam Inventory for Auditory Disability and Handicap; NFT: neurofibrillary tangle.

**Table 2:** Summary of studies on the relationship between HL and Aβ.

Author	Research Objects	Sample Size (N)	Age (Mean ± SD/Range, Year)	Indicators of Hearing	Indicators of Aβ	Result
Xu et al., 2019 [24]	Participants from ADNI	CSF Aβ42:479 Aβ PET: 666	CSF-HL group: 77.3 ± 5.2 CSF-HN group: 72.4 ± 6.9 PET-HL group: 77.3 ± 5.2 PET-HN group: 72.4 ± 6.9	MH and PE datasets	PET imaging and CSF Aβ42	ARHL was not associated with SUVRs or CSF Aβ42
Deal et al., 2023 [41]	Participants from ARIC	252	80.5 ± 5.0	PTA	Aβ PET imaging	Hearing was not associated with cortical or temporal lobe SUVRs
Sarant et al., 2022 [42]	Participants from AIBL	143	65.5–66.6	PTA	Aβ PET imaging	HL was not associated with brain Aβ once the age of individuals was controlled
Golub et al., 2021 [43]	Participants from NOMEM	98	64.6 ± 3.5	PTA or WRS	Aβ PET imaging	HL was independently associated with brain Aβ SUVRs
Van ’t Hooft et al., 2023 [44]	Oldest-old group: EMIF-AD 90+ study Younger-old group: EMIF-AD PreclinAD study	Oldest-old group:65 Younger-old group:60	oldest-old group: 92.7 ± 2.9 younger-old group: 74.4 ± 6.5	SRT	Aβ PET imaging	HL was associated with amyloid binding in younger-old individuals only

Note: SD: standard deviation; HL: hearing loss; ADNI: Alzheimer’s Disease Neuroimaging Initiative database; HN: hearing normal; MH: medical history; PE: physical exam; PET: positron emission tomography; CSF: cerebrospinal fluid; SUVRs: standardized uptake value ratios; ARHL: age-related hearing loss; ARIC: Atherosclerosis Risk in Communities-Positron Emission Tomography study data; PTA: pure-tone average; AIBL: Australian Imaging and Biomarker Longitudinal Study; NOMEM: The Northern Manhattan Study of Metabolism and Mind; WRS: word recognition score; EMIF-AD: European Medical Information Framework for AD project; SRT: speech reception threshold.

## Data Availability

No new data were created or analyzed in this study.

## Abbreviations

The following abbreviations are used in this manuscript:

HL	Hearing loss
ARHL	Age-related hearing loss
AD	Alzheimer’s disease
DMN	Default mode network
HAs	Hearing aids
NFTs	Neurofibrillary tangles
CSF	Cerebrospinal fluid
p-tau	Phosphorylated tau
t-tau	Total tau
PET	Positron emission tomography
MTL	Medial temporal lobe
Aβ	Amyloid beta-protein
CNS	Central nervous system
APP	Amyloid precursor protein
SUVRs	Standardized uptake value ratios
PTA	Pure-tone average
Glu	Glutamate
GABA	Gamma-aminobutyric acid
E/I	Excitation-inhibition
ROS	Reactive oxygen species
mtDNA	Mitochondrial DNA
VEGF	Vascular endothelial growth factor
BDNF	Brain-derived neurotrophic factor
pro-BDNF	Pro-brain-derived neurotrophic factor
m-BDNF	Mature brain-derived neurotrophic factor
GDF1	Growth/Differentiation factor 1
AEP	Asparagine endopeptidase
4-HNE	4-Hydroxynonenal
3-NT	3-Nitrotyrosine
TNF-α	Tumor necrosis factor-alpha
8-OHdG	8-Hydroxy-2-deoxyguanosine
NAC	N-Acetylcysteine
DHF	7,8-Dihydroxyflavone
TrkB	Tyrosine kinase receptor B
